# Predominance of Candida Glabrata among Non-*albicans* Candida Species in a 16-Year Study of Candidemia at a Tertiary Care Center in Lebanon

**DOI:** 10.3390/pathogens10010082

**Published:** 2021-01-19

**Authors:** Aline El Zakhem, Rachid Istambouli, Maria Alkozah, Amal Gharamti, Mohamad Ali Tfaily, Jean-Francois Jabbour, George F. Araj, Hani Tamim, Souha S. Kanj

**Affiliations:** 1 Department of Internal Medicine, American University of Beirut Medical Center, Beirut 1107 2020, Lebanon; az51@aub.edu.lb (A.E.Z.); ri51@aub.edu.lb (R.I.); Maria.Alkozah@hitchcock.org (M.A.); ae107@aub.edu.lb (A.G.); mht19@mail.aub.edu.lb (M.A.T.); jfjjabbour@stgeorgehospital.org (J.-F.J.); htamim@aub.edu.lb (H.T.); 2 Department of Pathology and Laboratory Medicine, American University of Beirut Medical Center, Beirut 1107 2020, Lebanon; garaj@aub.edu.lb

**Keywords:** invasive candidiasis, candidemia, non-*albicans Candida*, *Candida glabrata*, antifungal susceptibility, Lebanon, Arab world

## Abstract

Background: Candidemia is associated with a high mortality rate, and its incidence is increasing worldwide with a rise in non-*albicans* candidemia (NAC). Epidemiologic data from Arab countries are scarce and there are no data from Lebanon; Methods: This is a single-center retrospective chart review of patients with candidemia in a tertiary care center in Lebanon from 2004 to 2019. We extracted data on patient characteristics, isolated *Candida* species antifungal susceptibility, management and outcomes; Results: We included 170 cases of candidemia. NAC was more common than *albicans* candidemia (64.7% vs. 35.3%). *C. glabrata* was the most common non-*albicans* species (37 isolates) followed by *C. tropicalis* (14). Recent use of antifungals was a risk factor for NAC (OR = 2.8, *p* = 0.01), while the presence of a central venous catheter was protective (OR = 0.41, *p* = 0.02). Fluconazole resistance was 12.5% in *C. albicans* and 21.5% in non-*albicans* spp. Mortality at 30 days was 55.5%, with no difference between NAC and *albicans* candidemia. It was higher in older and critically ill patients but lower in patients whose central venous catheter was removed after detecting fungemia; Conclusions: Candidemia is associated with high mortality in Lebanon, with a predominance of NAC and high prevalence of *C. glabrata*.

## 1. Introduction

Candidemia is the most common form of invasive candidiasis, and is known to be the fourth to seventh most common bloodstream infection [1]. It is often associated with a high mortality rate of approximately 40–60% and a significant economic burden [2,3,4,5]. Due to the growing number of immunocompromised patients and critically ill patients, as well as the increasing use of invasive devices and antibiotics, the incidence of candidemia has been increasing around the world [2,6]. Particularly, there has been a rise in the incidence of non-*albicans* candidemia (NAC) [7].

Data on candidemia from Arab countries are limited [8]. In the absence of national surveillance programs, data are largely based on local studies. The crude mortality rate of candidemia in Arab countries is also elevated, and NAC is becoming more predominant as well [8]. In Lebanon, no studies have been previously done to evaluate the epidemiology of invasive candidiasis or candidemia. However, studies on the distribution of *Candida* spp. isolated from different sites without differentiation between colonization and true infection suggest an increasing incidence of non-*albicans Candida* and of fluconazole resistance [9,10].

Our aim is to identify the clinical characteristics of patients with candidemia, the isolated *Candida* spp. and their antifungal susceptibility, and the outcome of the infected patients. We also investigated the risk factors for NAC and increased mortality.

## 2. Results

### 2.1. Baseline Characteristics

Over the study period, we identified 193 cases of candidemia. We excluded 23 cases due to unavailability of full medical records. Almost half of the patients were female (49.4%) with a mean age of 65.7 (Table 1). Diabetes mellitus was a comorbid condition in 50 patients (29.4%) and 29 (17.1%) were on hemodialysis at infection onset, of which 19 (11.2%) were started on hemodialysis for acute kidney injury that occurred during the current admission.

At least one immunosuppressive factor was present in 110 patients (64.7%), malignancy being the most common (86 patients, 50.6%), followed by steroid intake (21.2%) and neutropenia (12.4%). A total of 38 patients had received chemotherapy in the month preceding the candidemia episode, of which 10 had received it in the one-week period prior to the infection onset. Other factors contributing to depressed immunity were immunotherapy (3), hematopoietic stem cell transplant (7), solid organ transplant (3), and congenital syndromes (3).

While 82 patients (48.2%) were admitted to a critical care unit at the onset of infection, 76 (44.7%) were on medical or surgical wards, and 12 (7.1%) were in the emergency department. Moreover, 83 patients (48.8%) were on mechanical ventilation, 44 (25.9%) were receiving parenteral nutrition, and 117 (68.8%) had a central venous catheter. Additionally, in the month prior to candidemia, 45 patients (26.5%) had undergone abdominal surgery, 161 (94.7%) had received antibiotics, and 45 (26.5%) had received antifungals.

A source of candidemia could be identified in 116 episodes (68.2%). The most common source was gastrointestinal translocation (33.5%), followed by Central Line Associated Bloodstream Infection (CLABSI) (23.5%) and urinary tract infection (10%). One case was the result of a vertebral abscess and another was caused by an aortoiliac graft infection.

### 2.2. Isolated Candida spp. and Antifungal Susceptibility

NAC (110 episodes, 64.7%) was more common than *C. albicans* candidemia (60 episodes, 35.3%) (Table 2). Speciation of non-*albicans Candida* was performed in 72 of 110 episodes of NAC. Of the speciated non-*albicans Candida*, *C. glabrata* was the most common (37), followed by *C. tropicalis* (14), *C. parapsilosis* (6), *Pichia kudriavzevii* (*C. krusei*) (5), *Kluyveromyces marxianus* (*C. kefyr*) (3), *Clavispora lusitaniaei* (*Candida lusitaniae*) (2), and *Wickerhamomyces anomalus* (*C. pelliculosa*) (1). There were four cases of mixed candidemia. *C. albicans* and *C. glabrata* were the two most commonly isolated species in all identified sources.

Results of the univariate analysis of possible NAC risk factors are shown in Table 3. Multivariate analysis (Table 4) showed that patients with a central venous catheter (CVC) were less likely to have NAC (OR = 0.41, *p* = 0.02), while a history of antifungal use within the last 30 days was associated with a higher likelihood of NAC (OR = 2.78, *p* = 0.01).

Antifungal susceptibility testing to fluconazole was performed on 89 isolates (Table 5). From a total of 24 *C. albicans* isolates tested, 12.5% were resistant to fluconazole. Non-*albicans Candida* displayed 21.5% resistance to fluconazole, mainly with *C. glabrata* (10/33) and *P. kudriavzevii* (*C. krusei*) (3/5). Resistance rates for voriconazole were 19.0% among tested *C. albicans* isolates, and 7.1% among tested non-*albicans Candida* isolates. All tested isolates were susceptible to caspofungin (14 isolates), and only 1 isolate of *C. glabrata* was resistant to amphotericin B (Table S1).

### 2.3. Management and Mortality

Data on mortality were available for 164 patients, and the 30-day crude mortality rate was 55.5%. Of those alive, candidemia recurred in 7 patients. Antifungals were administered to 142 patients (Table 6). An echinocandin (caspofungin, anidulafungin or micafungin) was started in 42.3% of cases, whereas fluconazole was started in 30% (dose of 400 mg daily was used in 16.5%). The mean treatment duration was 16.64 days, and 27.6% of patients received antifungals for more than 14 days after the first negative blood culture. Echocardiography was performed in 90 patients (52.9%), 5 of which had pertinent findings (3 had vegetations and 2 had new onset mitral valve regurgitation). Ophthalmic examination was performed on 35 patients (20.6%), 3 of which (8.6%) were found to have fungal endophthalmitis. Of 117 patients with CVC, 80 had the line removed after detection of candidemia (68.4%) and 67 (57.3%) had the catheter tip sent for culture.

A univariate analysis of mortality risk factors is presented in the supplementary table (Table S2). In the multivariate regression model (Table 7), mortality was significantly higher in older patients (OR = 1.04, *p* = 0.007), and in patients admitted to a critical care unit (OR = 3.92, *p* = 0.004). Conversely, patients for whom the central venous catheter was removed following the diagnosis of fungemia were less likely to be dead at 30 days (OR = 0.189, *p* = 0.021). There was no significant difference in the mortality between NAC (57.5%) and *C. albicans* candidemia (51.7%, *p* = 0.473).

## 3. Discussion

Although candidemia remains a cause of significant mortality and high economic burden worldwide, studies on candidemia in the Arab world remain limited, [8] with no data from Lebanon [11]. We report herein the first large series of candidemia at a tertiary hospital in Lebanon, detailing the characteristics, epidemiology, and outcomes of 170 candidemia episodes over a 16-year period.

Our study conforms with worldwide observations about the predominance of NAC [12], with 64.7% of the episodes caused by non-*albicans* species. Recent studies in the Arab countries were concordant with this global trend, reporting NAC rates of around 70% [8]. However, we found that *C. glabrata* is the most common causative agent for NAC in Lebanon (51.4% of speciated NAC). While this agrees with data from the United States, other regions such as Europe and Latin America have a much lower prevalence of *C. glabrata* [12]. This result also contrasts with data from neighboring countries in the Arab league, where *C. tropicalis* and *C. parapsilosis* were the most commonly reported causes of NAC, while *C. glabrata* represented only 0–19.2% of the cases [13,14,15,16,17]. *C. glabrata* is often associated with a higher mortality rate [18]. It is also inherently less susceptible to azole antifungals, with recent emergence of isolates resistant to echinocandins and amphotericin B [19,20], which highlights the importance of species identification and antifungal susceptibility testing in candidemia. Mixed candidemia was observed in four cases, and was associated with CLABSI three out of four times, a finding that was similarly reported in other studies [21]. Others have reported the effect of azole exposure on NAC emergence [22,23,24], which is consistent with our finding of antifungal use being associated with an almost threefold increase in the risk of developing NAC. We did not, however, find any association between NAC and other reported risk factors, such as immunosuppression, parenteral nutrition, old age, and critical illness [25]. An important finding in our study was that patients with CVC were less likely to develop NAC (OR = 0.48, *p* = 0.048), which contradicts other studies [26].

Although *C. albicans* is generally considered susceptible to fluconazole, reports of *C. albicans*’ sensitivity to fluconazole are widely variable in the literature (35–100%) [13,27,28]. Among the 24 *C. albicans* isolates in our cohort, 3 (12.5%) were resistant to fluconazole. None of these three patients had received antifungal therapy in the month preceding the candidemia, and all three had a solid malignancy and died within 30 days. An additional *C. albicans* isolate was also resistant to voriconazole. Among non-*albicans* isolates, *P. kudriavzevii* (*C. krusei*) displayed the highest resistance to fluconazole (60%), followed by *C. glabrata* (30.3%). *C. tropicalis* was resistant to fluconazole and to voriconazole in 7.1% and 8.3% of the tested isolates, respectively. This highlights the clinical significance of NAC, as these species have a widely reported resistance to azoles. Only 1 *C. glabrata* isolate was resistant to amphotericin B, and all tested isolates (14) were susceptible to caspofungin. In light of these results, the practice of empiric therapy of candidemia with echinocandin followed by step-down therapy to fluconazole in susceptible isolates is justified by our results [29].

In our cohort, 30-day mortality was 55.5%, falling in the range of crude mortality reported elsewhere (40–60%) [3,4,5,8]. Risk factors associated with higher mortality are not uniform among studies, with some characteristics reported including intensive care unit stay, CLABSI, septic shock [30], advanced age [31], acute kidney injury, malignancy [8], and neutropenia [32]. While NAC is associated with increased mortality in some series [27], it is associated with decreased mortality in others [33]. There was no statistically significant difference in mortality between NAC and *albicans* candidemia in our cohort (OR = 1.3, *p* = 0.473). However, older patients in our study and patients admitted to a critical care unit had a higher 30-day mortality.

Candidemia management guidelines are evolving, and some recommendations remain controversial [29,34]. In our center, echinocandins were the most common first-line antifungals used, which is consistent with the current guidelines [29]. Moreover, concerning the controversies over central venous catheter removal following the diagnosis of candidemia [34], we show in our cohort that this approach was associated with decreased mortality.

This study has a few limitations, including its retrospective nature. Medical records were not available for 23 candidemia cases identified in the Electronic Health Records. We also did not exclude terminal patients, in whom antifungal therapy was withheld due to patient/family wishes for a palliative care approach. Moreover, not all non-*albicans* isolates were speciated and tested for antifungal susceptibility, particularly among the isolates from the earlier years of the study.

## 4. Materials and Methods

We conducted a retrospective chart review at the American University of Beirut Medical Center (AUBMC), a tertiary care center in Lebanon, over a span of 16 years (2004–2019). AUBMC is the largest academic tertiary care center in Lebanon with a busy oncology unit including a stem cell transplant service, and medical and surgical intensive care units receiving patients from across Lebanon as well as referrals from neighboring countries. Using the Electronic Health Records at our institution, we identified all occurrences where *Candida* species grew in blood cultures. We included all episodes of candidemia occurring in patients ≥18 years old. Recurrence was considered when two episodes of candidemia occurred ≥7 days apart with clinical and microbiological resolution in the interim. We excluded cases where medical records were not available. We excluded from the mortality analysis cases where the outcome at 30 days was not available.

We reviewed the medical records of all cases and extracted data on demographics (age, sex), comorbid conditions (diabetes mellitus, hemodialysis), immunosuppression (malignancy, chemotherapy, or immunotherapy within the previous 30 days, hematopoietic or solid organ transplantation, neutropenia, steroid exposure, congenital, or acquired immunodeficiency syndromes), and patient characteristics at the onset of infection (hospital unit, mechanical ventilation, presence of CVC, parenteral nutrition, and abdominal surgery within the previous 30 days, antibiotic and antifungal history within the previous 30 days, source of candidemia). We defined neutropenia as an absolute neutrophil count (ANC) <1500 cells/mm^3^ which we classified as mild (<1500 and ≥1000 cells/mm^3^), moderate (<1000 and ≥500 cells/mm^3^), or severe (<500 cells/mm^3^) [35]. We defined steroid exposure as intake of the equivalent of 20 mg of prednisone for more than 1 month. We used the National Healthcare Safety Network definition of CLABSI [36]. We classified an episode as NAC whenever a non-*albicans Candida* species was recovered from the initial blood culture, whether it occurred alone or as a co-infection with *Candida albicans*.

Blood culture bottles were incubated in a BACT/ALERT^®^ system (Durham, North Carolina, NC, USA), and *Candida* species identification and antifungal susceptibility testing were done using VITEK^®^ 2 system (BioMérieux, Marcy L’Etoile, France). Antifungal susceptibility was reported using the Clinical and Laboratory Standards Institute criteria [37].

We analyzed data using IBM SPSS version 22 (IBM, New York, NY, USA). We described continuous data using mean and standard deviation, and we used the Student *t*-test for comparison between groups. For categorical data, we used the Pearson chi-square test or Fischer’s exact test for comparison. Multivariate regression was used to investigate risk factors for NAC and 30-day mortality using a stepwise approach. Risk factors with *p*-value < 0.05 in univariate analysis were included in the model. Predictors with *p*-values < 0.05 were considered as statistically significant.

## 5. Conclusions

Candidemia is associated with a high mortality in Lebanon, irrespective of the causative species. NAC is more common than *albicans* candidemia, with *C. glabrata* being the leading cause of non-*albicans* infections. These local epidemiological data are of paramount importance for the development of optimal treatment strategies and tackling current controversies in management.

## Figures and Tables

**Table 1:** Baseline characteristics of the patients with candidemia, including demographics, comorbid conditions, immunosuppression, and admission characteristics.

Characteristics	n (%)	
**Demographics and Comorbidities**		
Age (years, mean ± SD)	65.71	±17.175
Female	84	(49.4)
Diabetes mellitus	50	(29.4)
Hemodialysis	29	(17.1)
End-stage renal disease	10	(5.9)
Acute kidney injury	19	(11.2)
**Immunosuppression**		
Malignancy	86	(50.6)
Non-hematologic	62	(36.5)
Hematologic	20	(11.8)
Both	4	(2.4)
Chemotherapy	38	(22.4)
<1 week from infection	10	(5.9)
Immunotherapy	3	(1.8)
Hematopoietic stem cell transplant recipient	7	(4.1)
Auto-transplant	3	(1.8)
Allo-transplant	3	(1.8)
Both	1	(0.6)
Neutropenia	21	(12.4)
1000–1500/mm^3^	3	(1.8)
500–1000/mm^3^	6	(3.5)
<500/mm^3^	12	(7.1)
Current steroids	36	(21.2)
Solid organ transplant recipient	3	(1.8)
Congenital syndromes	3	(1.8)
Other immunosuppressants	9	(5.3)
**Admission Characteristics**		
Unit		
Regular ward	76	(44.7)
Critical care unit	82	(48.2)
Emergency department	12	(7.1)
Abdominal surgery within 30 days	45	(26.5)
Antibiotics use within 30 days	161	(94.7)
Antifungals use within 30 days	45	(26.5)
Fluconazole	31	(18.2)
Voriconazole	2	(1.2)
Lipid formulation of amphotericin B	4	(2.4)
Caspofungin	8	(4.7)
Anidulafungin	2	(1.2)
Duration of previous antifungal (days, mean ± SD)	13.43	±15.66
Mechanical ventilation	83	(48.8)
Parenteral nutrition	44	(25.9)
Central venous catheter	117	(68.8)
Source		
Gastrointestinal translocation	57	(33.5)
CLABSI	40	(23.5)
Urinary tract infection	17	(10)
Vertebral abscess	1	(0.6)
Aortoiliac graft infection	1	(0.6)
Unknown	54	(31.8)

Data are presented as number (percentage) unless otherwise specified. CLABSI: Central Line-Associated Bloodstream Infection.

**Table 2:** Species distribution of *Candida* isolates.

*Candida* Species	n
*Candida albicans*	60
*Candida* non-*albicans*	110
*C. glabrata*	37
*C. tropicalis*	14
*C. parapsilosis*	6
*P. kudriavzevii* (*C. krusei*)	5
*K. marxianus* (*C. kefyr*)	3
*C. lusitaniae*	2
*W. anomalus* (*C. pelliculosa*)	1
*C. albicans* and *C. glabrata*	1
*C. albicans* and *C. tropicalis*	1
*C. albicans* and *C. dubliniensis*	1
*C. albicans* and *C. parapsilosis*	1
unspeciated	38
Total	170

**Table 3:** Univariate analysis of possible risk factors associated with non-*albicans* candidemia. Data are presented as number (percentage) unless otherwise specified.

Variable	*C. albicans*	*C.* non-*albicans*	*p*-Value
Age (years, mean ± SD)	68.55 ± 15.5	64.15 ± 17.9	0.111
Female	26 (43.3)	58 (52.7)	0.242
Year of admission			
2004–2009	10 (16.7)	20 (18.2)	0.313
2010–2014	26 (43.3)	35 (31.8)	
2015–2019	24 (40.0)	55 (50.0)	
Unit			
Regular ward	23 (38.3)	53 (48.2)	0.418
Critical care unit	33 (55.0)	49 (44.5)	
Emergency department	4 (6.7)	8 (7.3)	
Diabetes mellitus	17 (28.3)	33 (30.0)	0.820
Hemodialysis patient	13 (21.7)	16 (14.5)	0.238
End-stage renal disease	6 (10.0)	4 (3.6)	0.169
Acute kidney injury	7 (11.7)	12 (10.9)	0.881
Solid organ transplant recipient	0 (0.0)	3 (100)	0.553
Stem cell transplant recipient	1 (1.7)	6 (5.5)	0.423
Auto-transplant	1 (100)	2 (33.3)	0.459
Allo-transplant	0 (0.0)	3 (50.0)	
Both	0 (0.0)	1 (16.7)	
Neutropenic	6 (10.0)	15 (13.6)	0.628
1000–1500/mm^3^	2 (33)	1 (6.7)	0.219
500–1000/mm^3^	2 (33)	4 (26.7)	
<500/mm^3^	2 (33)	10 (66.7)	
Immunocompromised	38 (63.3)	72 (65.5)	0.782
Current steroids	14 (23.3)	22 (20.0)	0.611
Congenital syndromes	2 (3.3)	1 (0.9)	0.285
Other immunosuppressants	2 (3.3)	7 (6.4)	0.495
Malignancy	27 (45.0)	59 (53.6)	0.282
Non-hematologic	21 (77.8)	41 (69.5)	0.729
Hematologic	5 (18.5)	15 (25.4)	
Both	1 (3.7)	3 (5.1)	
Chemotherapy	11 (18.3)	27 (24.5)	0.353
<1 week from infection	3 (27.3)	7 (25.9)	1.00
Immunotherapy	0 (0.0)	3 (2.7)	0.553
Abdominal surgery within 30 days	16 (26.7)	29 (26.4)	0.966
Antibiotics within 30 days	55 (91.7)	106 (96.4)	0.191
Antifungal history	10 (16.7)	35 (31.8)	0.032 *
Fluconazole	6 (10.0)	25 (22.7)	0.040 *
Voriconazole	0 (0.0)	2 (1.8)	0.541
Amphotericin B	1 (1.7)	3 (2.7%)	1.00
Caspofungin	3 (5.0)	5 (4.5)	1.00
Anidulafungin	0 (0.0)	2 (1.8)	0.541
Duration of previous antifungal (days, mean ± SD)	16.6 ± 24.8	16.7 ± 27.3	0.989
Parenteral nutrition	16 (26.7)	28 (25.5)	0.863
Central venous catheter	47 (78.3)	70 (63.6)	0.048 *
Source			
CLABSI	18 (30.0)	22 (20.0)	0.142
Gastrointestinal translocation	18 (30.0)	39 (35.5)	0.472
Urinary tract infection	6 (10.0)	11 (10.0)	1.00
Unknown	18 (30.0)	36 (32.7)	0.715
Other	0 (0.0)	2 (1.8)	0.528

CLABSI: Central Line-Associated Bloodstream Infection. *: *p*-values < 0.05.

**Table 4:** Multivariate regression for predictors of non-*albicans* candidemia.

Variable	OR (95% CI)	*p*-Value
Central Venous Catheter	0.41 (0.19–0.86)	0.02
Antifungal History	2.78 (1.23–6.25)	0.01

Variables included in the model were central venous catheter, history of antifungal use and history of fluconazole use. OR: Odds Ratio.

**Table 5:** Antifungal susceptibility of *Candida* isolates to fluconazole and voriconazole. % resistant: percentage of isolates resistant to antifungal.

	Fluconazole					Voriconazole				
*Candida* Species	n	Sensitive	Intermediate	Resistant	% Resistant	n	Sensitive	Intermediate	Resistant	% Resistant
*C. albicans*	24	21	0	3	12.5%	21	17	0	4	19.0%
*C. glabrata*	33	21	2	10	30.3%	28	23	2	3	10.7%
*C. tropicalis*	14	13	0	1	7.1%	12	11	0	1	8.3%
*C. parapsilosis*	7	7	0	0	0.0%	5	5	0	0	0.0%
*P. kudriavzevii* (*C. krusei*)	5	1	1	3	60.0%	5	5	0	0	0.0%
*K. marxianus* (*C. kefyr*)	3	3	0	0	0.0%	3	3	0	0	0.0%
*C. lusitaniae*	2	2	0	0	0.0%	2	2	0	0	0.0%
*W. anomalus* (*C. pelliculosa*)	1	1	0	0	0.0%	1	1	0	0	0.0%
Total	89	69	3	17	19.1%	77	67	2	8	10.4%

**Table 6:** Management of candidemia.

Management	n (%)	
Species identified	132	(77.6)
Echocardiography	90	(52.9)
Ophthalmic exam	35	(20.6)
Empiric therapy		
Fluconazole	51	(30.0)
Caspofungin	41	(24.1)
Anidulafungin	18	(10.6)
Micafungin	13	(7.6)
Lipid formulation of amphotericin B	12	(7.1)
Itraconazole	5	(2.9)
Voriconazole	2	(1.2)
Empiric therapy changed	62	(36.5)
Targeted therapy		
Fluconazole	31	(18.2)
Voriconazole	6	(3.5)
Caspofungin	10	(5.9)
Anidulafungin	3	(1.8)
Micafungin	3	(1.8)
Lipid formulation of amphotericin B	8	(4.7)
Other	1	(0.6)
Antifungal duration (days, mean ± SD)	16.64	±26.334
Antifungal at least 14 days after first negative culture	47	(27.6)
Central venous catheter removed	80	(47.1)
Tip sent for culture	67	(39.4)

**Table 7:** Multivariate regression for predictors of 30-day mortality.

Variable	OR (95% CI)	*p*-Value
Age	1.04 (1.01–1.07)	0.007
Admission to critical care units	3.92 (1.54–9.94)	0.004
Central venous catheter removal	0.189 (0.046–0.779)	0.021

Variables included in the model were: age; neutropenia; unknown source; echocardiography; ophthalmic exam; first antifungal anidulafungin; central venous catheter removed; critical care. OR: Odds Ratio.

## Data Availability

The data presented in this study are available on request from the corresponding author. The data are not publicly available due to privacy.

## Supplementary Materials

The following are available online at https://www.mdpi.com/2076-0817/10/1/82/s1, Table S1: Antifungal susceptibility of *Candida* isolates to amphotericin B and caspofungin, Table S2: Univariate analysis of possible risk factors associated with 30-day mortality.
