# Supplementary material for: Predominance of Candida Glabrata among Non-albicans Candida Species in a 16-Year Study of Candidemia at a Tertiary Care Center in Lebanon
Source: Pathogens. 2021 Jan 19;10(1):82. doi: 10.3390/pathogens10010082 (PMC7832319; doi:10.3390/pathogens10010082)
Supplement: Supplementary file 1 [file pathogens-10-00082-s001.pdf]

Table S1. Antifungal susceptibility of *Candida* isolates to amphotericin B and caspofungin. *%resistant: percentage of isolates resistant to antifungal.*

	Amphotericin B					Caspofungin			
Candida species	n	Sensitive	intermediate	resistant	% resistant	n	sensitive	resistant	%resistant
C. albicans	20	20	0	0	0.0%	5	5	0	0.0%
C. glabrata	20	19	0	1	5.0%	5	5	0	0.0%
C. tropicalis	8	8	0	0	0.0%	2	2	0	0.0%
C. parapsilosis	5	5	0	0	0.0%	1	1	0	0.0%
P. kudriavzevii (*C. krusei*)	2	2	0	0	0.0%	0	0	0	0.0%
K. marxianus (*C. kefyr*)	2	2	0	0	0.0%	1	1	0	0.0%
C. lusitaniae	1	0	1	0	0.0%	0	0	0	0.0%
Total	58	56	1	1	1.7%	14	14	0	0.0%

Table S2. Univariate analysis of possible risk factors associated with 30-day mortality. Data are presented as percentages unless otherwise specified. *CLABSI: Central Line-Associated Bloodstream Infection. *:p-values<0.05*

**Characteristic**		**Alive at 30 days**	**Dead at 30 days**	**p-value**
Age (years, mean ± SD)		61.4 ± 18.6	70 ± 14.9	**0.002***
Female		47.90	49.50	0.848
Year of admission				
	2004-2009	19.2	15.4	
	2010-2014	45.2	30.8	0.062
	2015-2019	35.6	53.8	
Critical care unit		27.4	64.8	**<0.001***
Diabetes mellitus		27.4	30.8	0.637
Hemodialysis patient		13.7	20.9	0.231
	End-stage renal disease	6.8	5.5	0.753
	Acute kidney injury	6.8	15.4	0.09
Solid organ transplant recipient		0.0	2.2	0.503
Stem cell transplant recipient		4.1	4.40	1.00
	Auto-transplant	33.3	50.0	
	Allo-transplant	33.3	50.0	1.00
	Both	33.3	0.0	
Neutropenic		6.8	17.6	**0.041***
	1000-1500/mm^3	20.0	12.5	
	500-1000/mm^3	0.0	37.5	0.351
	<500/mm^3	80.0	50.0	
Immunocompromised		56.2	70.3	0.060
Current steroids		16.4	25.3	0.170

Congenital syndromes	2.7	1.1	0.586
Other immunosuppressants	2.7	6.6	0.301
Malignancy	46.6	53.8	0.355
Non-hematologic	23.5	24.5	
Hematologic	73.5	69.4	0.926
Both	2.9	6.1	
Chemotherapy	24.7	22.0	0.686
<1 week from infection	33.3	20.0	0.468
Immunotherapy			
Abdominal surgery within 30 days	31.5	23.1	0.226
Antibiotics within 30 days	95.9	93.4	0.732
Antifungal history	23.3	28.6	0.445
Fluconazole	19.2	18.7	0.936
Voriconazole	1.4	1.1	1.00
Amphotericin B	1.4	3.3	0.63
Caspofungin	1.4	5.5	0.227
Anidulafungin	0.0	2.2	0.503
Duration of previous antifungal (days, mean ± SD)	9.13 ± 12.2	16.7 ± 17.9	0.152
Parenteral nutrition	27.4	25.3	0.759
Central venous catheter	63.0	75.8	0.075
Source			
CLABSI	27.4	19.8	0.251
Gastrointestinal translocation	35.6	33.0	0.722
Urinary tract infection	13.7	6.6	0.128
Unknown	19.2	38.5	**0.007***
Other	1.4	1.1	1.00
Candida non-albicans	61.6	63.7	0.783
Echocardiography	63.0	46.2	**0.031***
Ophthalmic exam	28.8	14.3	**0.023***
Empiric therapy			
Fluconazole	40.6	30.9	0.236
Voriconazole	1.4	1.5	1.00
Itraconazole	4.3	2.9	1.00
Caspofungin	27.5	29.4	0.808
Anidulafungin	7.2	19.1	**0.04***
Micafungin	10.1	8.8	0.792
Lipid formulation of amphotericin B	8.7	7.4	0.772
Central venous catheter removed	91.3	53.6	**<0.001 ***
Tip sent for culture	88.1	78.4	0.245
